# Evaluation of a Novel Artificial Intelligence System to Monitor and Assess Energy and Macronutrient Intake in Hospitalised Older Patients

**DOI:** 10.3390/nu13124539

**Published:** 2021-12-17

**Authors:** Ioannis Papathanail, Jana Brühlmann, Maria F. Vasiloglou, Thomai Stathopoulou, Aristomenis K. Exadaktylos, Zeno Stanga, Thomas Münzer, Stavroula Mougiakakou

**Affiliations:** 1 ARTORG Center for Biomedical Engineering Research, University of Bern, Murtenstrasse 50, 3008 Bern, Switzerland; ioannis.papathanail@unibe.ch (I.P.); maria.vasiloglou@unibe.ch (M.F.V.); thomai.stathopoulou@unibe.ch (T.S.); 2 Geriatrische Klinik St. Gallen AG, Rorschacherstrasse 94, 9000 St. Gallen, Switzerland; jana.bruehlmann@students.unibe.ch (J.B.); thomas.muenzer@geriatrie-sg.ch (T.M.); 3 Department of Emergency Medicine, Bern University Hospital, University of Bern, 3010 Bern, Switzerland; aristomenis.exadaktylos@insel.ch; 4 Department of Diabetes, Endocrinology, Nutritional Medicine and Metabolism, Inselspital, Bern University Hospital, University of Bern, 3010 Bern, Switzerland; zeno.stanga@insel.ch

**Keywords:** dietary assessment, artificial intelligence, dietary intake, geriatrics, malnutrition

## Abstract

Malnutrition is common, especially among older, hospitalised patients, and is associated with higher mortality, longer hospitalisation stays, infections, and loss of muscle mass. It is therefore of utmost importance to employ a proper method for dietary assessment that can be used for the identification and management of malnourished hospitalised patients. In this study, we propose an automated Artificial Intelligence (AI)-based system that receives input images of the meals before and after their consumption and is able to estimate the patient’s energy, carbohydrate, protein, fat, and fatty acids intake. The system jointly segments the images into the different food components and plate types, estimates the volume of each component before and after consumption, and calculates the energy and macronutrient intake for every meal, based on the kitchen’s menu database. Data acquired from an acute geriatric hospital as well as from our previous study were used for the fine-tuning and evaluation of the system. The results from both our system and the hospital’s standard procedure were compared to the estimations of experts. Agreement was better with the system, suggesting that it has the potential to replace standard clinical procedures with a positive impact on time spent directly with the patients.

## 1. Introduction

Malnutrition in hospitalised patients is a serious condition with significant consequences on all organ systems. Malnutrition according to the European Society for Clinical Nutrition and Metabolism (ESPEN) guideline, can be defined as “a state resulting from lack of intake or uptake of nutrition that leads to altered body composition and body cell mass leading to diminished physical and mental function and impaired clinical outcome from disease” [1]. Studies on the occurrence of malnutrition in hospitalised patients have demonstrated that between 20–60% of patients admitted to hospitals in western countries [2] and 20–30% of patients admitted to hospitals in Switzerland [3] were malnourished. Especially, older patients are a high-risk population for developing undernutrition or malnutrition [4]. Several studies have reported that geriatric patients who suffer from malnutrition have an increased risk of longer hospital stays and higher mortality and longer rehabilitation periods [5,6,7,8,9,10]. Although the serious impacts of malnutrition in older people are well-known, it is often under-recognised, thus underdiagnosed and consequently often remains untreated [5]; therefore, it is highly important to correctly identify and treat malnourished patients in acute geriatric hospitals [11].

Treatment of malnutrition is very time-consuming and requires individualised treatment plans. However, recent studies have demonstrated that such treatment plans are effective; the Nourish [12] and the EFFORT [13] studies both demonstrated that nutritional support of malnourished patients in geriatrics significantly reduces the risk of adverse outcomes and mortality [14]. Increased malnutrition risk during hospitalisation is predominantly attributed to the poor monitoring of nutritional intake [15]. Regular evaluation of food intake in hospitalised patients is therefore recommended to lower the risk of malnutrition and, thus, to improve nutritional intake to positively influence the clinical outcomes as well as reduce health care costs [16]. In addition, to accurately assess nutritional intake, the estimation of food leftovers (volume or percentage of food discarded) is crucial.

One important but time-consuming aspect of malnutrition treatment is monitoring the patients’ food intake. In order to record food intake, manual food or plate protocols can be used to document the amount of food consumed by the patient. The current gold standard for the estimation of consumed food is the weighing of patient’s meals before and after consumption and the calculation of energy and nutrient intake based on weight difference [12]. Since weighing individual meals is very demanding, different studies have used visual food protocols where trained personnel estimated the food consumed, which can be used as a clinically appropriate tool to measure energy and protein intake instead of weighing the meals [12,17,18,19,20,21]. Several studies validated the use of direct visual estimation and indirect visual estimation by photography [18,19] as appropriate replacement methods. In contrast, other studies have reported that visual estimation protocols significantly differ from the weighing method, especially for size-adjusted meals and the protein intake of the patients [21,22]. In addition, food protocols are hardly integrated into daily clinical practice, which is primarily because the estimation process is time-consuming and is subjective to the perception of the nursing staff that is responsible for the procedure [23]. The usage of such protocols might be increased, by using digital food protocols, enhanced by Artificial Intelligence (AI)-based algorithms that can automatically calculate the food consumed by the patients on the basis of food-image analysis from pictures taken before the food is served and after its consumption.

In our previous publication [24], we developed an AI-based system that automatically estimates the energy and nutrient consumption of hospitalised patients by processing one Red, Green, Blue-Depth (RGB-D) image before and one after meal consumption. The results demonstrated that such an automatic system could be highly convenient in a hospital environment.

The aim of this study is to evaluate the performance of our AI-based system for the estimation of energy and macronutrient intake in hospitalised patients and compare its performance with the standard clinical procedure in a geriatric acute care hospital in St. Gallen, Switzerland. As the reference method, the visual estimations of two experts in nutrition and a trained medical student were used. In comparison to our previous publication [24], food segmentation and recognition are optimised and improved, and we also focus on geriatric acute care hospitalised patients. To analyse and assess the performance of our refined system in geriatrics, we compared the estimation performed by the system with the estimations of the nursing staff following the hospital’s standard procedure, against the visual estimations of two experienced dietitians and a trained medical student.

## 2. Materials and Methods

### 2.1. Data Collection

**Figure 1:**
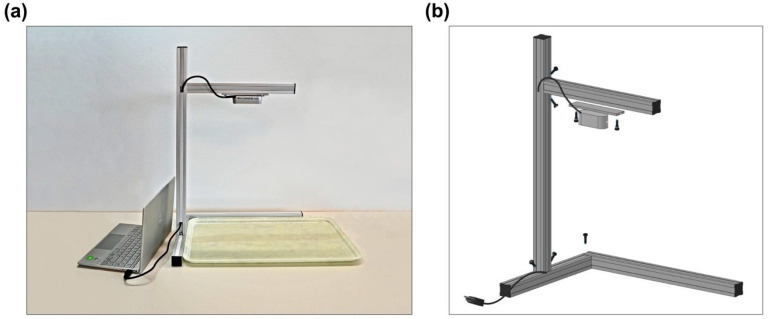
Standardised mount for capturing images by the RGB-D camera; (**a**) setup for image capture—the RGB-D camera is connected to a laptop and on top of an empty tray; (**b**) expanded view of the mount showing the mechanical parts of the mount for the RGB-D camera.

This pilot study was conducted at the Geriatrische Klinik in St. Gallen, Switzerland, between 14 December 2020, and 12 February 2021. The Intel RealSense Depth Camera D455i was used to capture the meal images, including both the RGB and depth images of the meals (Width × Height: 640 pixels × 480 pixels), before and after their consumption by the patients. Since this technology also measures depth data, a fiducial marker was not needed. In order to obtain comparable pictures for the analysis by the automatic system, a mount for the RGB-D camera was developed and built by the hospital’s technical department using commercially available metal (aluminium) profiles, (Figure 1). All the meals were served on a standardised tray. In order to allow the acquisition of the whole serving tray, the camera was placed at the height of 47 cm.

**Figure 2:**
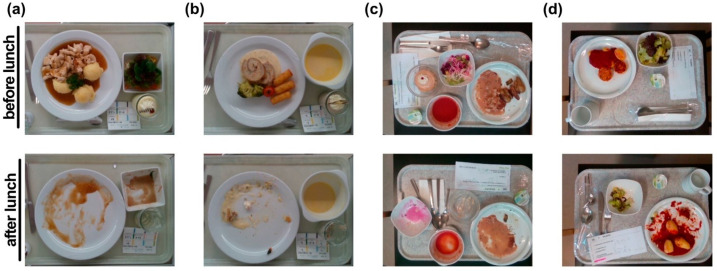
Sample images before and after meal consumption from Geriatrische Klinik St. Gallen (**a**,**b**) and from the Bern University Hospital [24] (**c**,**d**).

The typical procedure for capturing the image and serving the meal was as follows. First, a trained medical student used the mounted RGB-D camera to capture an image of the respective meal before consumption, which was then served to the patient. No time limit was given to the patient to finish the meal. After the patient had finished the meal, the same medical student took an “after” image using the camera sensor. Each image was assigned an individual number referring to the patient, the meal composition, meal size, and the date and time of acquisition. Examples of meal images before and after consumption can be seen in Figure 2a,b.

In total, 166 meals (332 images) on 32 weekdays from 28 patients were recorded. Inclusion criteria for the study were that the patients were able to choose their lunch menu independently and were able to eat the meals by themselves. For this study, only lunch meals were assessed in two separate patient rooms that were defined prospectively.

Additionally, the newly acquired database was enhanced by images from our previous study [24]. Even though the study has been localised in a specific hospital, we investigated if data from another hospital environment may enhance the system’s performance. The database from our previous study consists of 534 images depicting meals prepared by the Bern University Hospital (Figure 2c,d).

### 2.2. Dietary Meal Analysis

Each meal was selected by the patients, with the assistance of the nursing staff, on the basis of doctors’ prescription in accordance with the Nutritional Risk Screening (NRS) [25] and the patient’s food tolerances. The patients could choose between four different meal sizes: normal, increased (4/3 of the normal meal size), reduced (2/3), and doubly reduced (1/3) meal size. Of the total of 166 meals that were recorded, 51 (30.7%) were normal size, and only 6 (3.6%) of increased size. The numbers of reduced and doubly reduced meals were 85 (51.2%) and 24 (14.5%), respectively.

**Figure 3:**
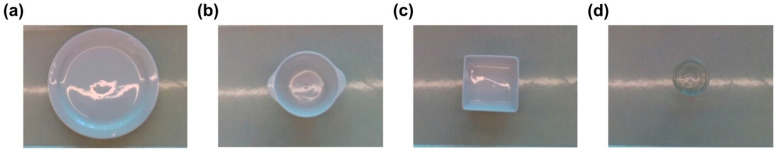
The plates used in the Geriatrische Klinik, St. Gallen: (**a**) round plate for main course; (**b**) bowl for soup; (**c**) square bowl for salad or dessert; (**d**) glass for dessert.

Each menu was composed of a soup, a main course, and a dessert. The normal main course consisted of up to four components: meat or fish, a side dish, sauce, and vegetables or a salad. A vegetarian main course consisted of a vegetarian main dish or only the side dish instead of the meat or fish dish. Each meal was also available in a soft homogenous or mashed version. The soup and the dessert were usually supplemented with protein powder (protein+) to meet the recommended protein intake of geriatric patients. There were four different plates that were used to serve the food, i.e., round plate, soup bowl, square bowl, and glass for dessert (Figure 3). A typical lunch menu with three different meal compositions is depicted in Table 1.

In order to monitor the calorie and macronutrient intake for each patient, the percentage of consumed food was first estimated independently by people of different scientific backgrounds: The trained medical student, two dietitians, and the instructed nursing staff. The trained medical student estimated the consumed food by the patient after it had been cleared away. The two dietitians used the “before” and “after” meal images for their visual estimations. The nursing staff estimated the consumed food by the patient after it had been cleared away following their typical procedure of the kitchen: i.e., estimation of the total food consumed in the 10% scale without taking into account the different dishes (e.g., 40% of the total food was consumed). The experienced dietitians and the trained medical student estimated the food consumed separately for each component of the meal in the 10% and 25% scales.

In this preliminary study, the weight of the dishes and the meal components was not measured before and after consumption and, thus, the ground truth (GT) could not be obtained by this procedure. For this reason, the mean of the estimations of the two dietitians and the trained medical student for the consumed percentage of each meal component was used as a reference and defined as visual estimations (reference) due to a high correlation with weighing [12,17,18,19,20,21].

**Figure 4:**
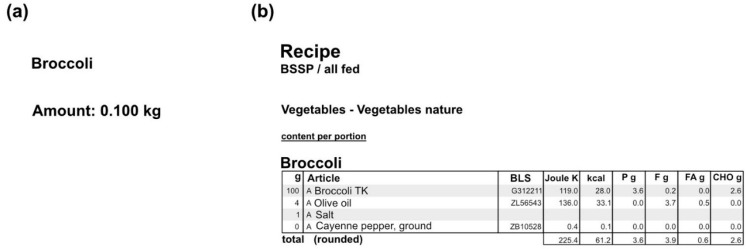
An example of exported pdf file from the kitchen database for the dish “Broccoli”: (**a**) the quantity of a normal-sized dish; (**b**) the macronutrients of a normal-sized dish (P: protein, F: fat, FA: fatty acids, CHO: carbohydrates).

In order to calculate the energy and macronutrient intake of the food consumed by the patients, the nutritional information of the individual menus was retrieved from the kitchen database (SANALOGIC Solutions GmbH [26]). For each meal, we extracted the quantity of the individual components and the macronutrients of a normal-sized dish and exported as PDFs, as depicted in Figure 4. The respective quantity of the food items was measured in litres, kilograms, pieces, or portions. The macronutrients that are being used for the study, along with the energy (kcal), are carbohydrates (CHO), protein, total fat, and fatty acids (all measured in grams—g). For each dish, the respective quantity and macronutrients were multiplied by the size of the meal that each patient chose, in order to estimate the total ones.

### 2.3. Food Segmentation Network

**Figure 5:**
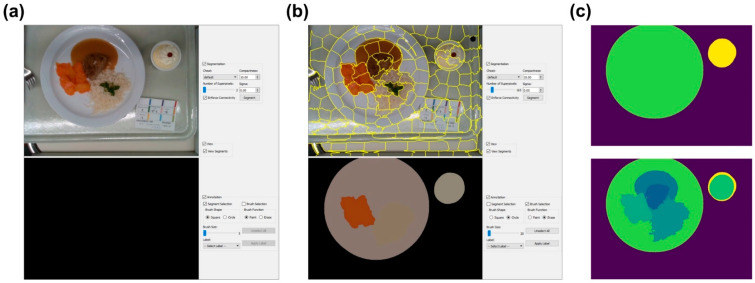
The segmentation tool that was used to provide the ground truth of the segmentation (GT_seg_): (**a**) the interface of the segmentation tool; (**b**) the semi-automatic segmentation; (**c**) the segmented plates of the images (up) and food types (bottom).

In order to create a system that can accurately estimate the energy and macronutrient intake from the RGB-D images before and after the meal consumption, a segmentation network needs to be trained first. A segmentation network receives as its input a single RGB image and partitions it into different elements. In this project, it is needed to segment the pictures into the six food types (soup, meat/fish, side dish, sauce, vegetables/salad, dessert), the four plate types (round plate, soup bowl, square bowl, glass) and the background. To obtain the GT of the segmentation (GT_seg_) for the 332 images, we used a semi-automatic segmentation tool that was developed by our team (Figure 5). The tool automatically provides a segmentation mask for each image, which can then be refined and adjusted by the user.

For the segmentation network, we experimented with two network architectures: The Pyramid Scene Parsing Network (PSPNet) [27] and the DeepLabv3 [28] architecture.

For the PSPNet, a convolutional neural network (CNN) was firstly used to extract the feature map for the image (size of 30 × 40 × 2048). For the CNN, we used either the ResNet50 [29] architecture (ResNet + PSPNet) or a simple encoder with five stacks of convolutional layers in a row (Encoder + PSPNet). A pyramid parsing module was applied to the feature map to identify features in four different scales (1 × 1 × 2048, 2 × 2 × 2048, 3 × 4 × 2048, and 6 × 8 × 2048). The four new feature maps were then upsampled to 30 × 40 × 512 and concatenated with the original feature map. A deconvolutional layer was applied in order to resize the maps to the original size of the image. This procedure was implemented twice, for the plate and the food segmentation, and the outputs received were 480 × 640 × 5 (four different plates and background) and 480 × 640 × 8 (six meal-dishes, plate, and background) in size.

Similarly, for the DeepLabv3, the ResNet50 CNN was used to extract the feature map. An Atrous Spatial Pyramid Pooling is then added on top of the feature map, which performs (a) a 1 × 1 convolution, (b) three 3 × 3 convolutions with different dilation rates (the dilation rate adapts the field-of-view of the convolution), and (c) an image pooling module to include global information. The results are then concatenated, convoluted with kernel 1 × 1, and upsampled to obtain the two outputs for the plate and the food segmentation.

Both the networks were trained with the “Adadelta” optimiser and a batch size equal to 8, for up to 100 epochs. We also experimented by adding and removing the plate segmentation module in the architecture.

From the 332 images in total, 292 were used for training of the segmentation network and 40 images (20 before and 20 after consumption) for testing. For the segmentation network, we compared the PSPNet and the DeepLabv3 architectures with ResNet as the backbone (with and without the module for plate segmentation) and the PSPNet with the simple encoder as the backbone network. In order to evaluate the performance of the segmentation network, the following metrics were used: (a) the mean Intersection over Union (mIoU), which is the mean of the intersection of GT_seg_ pixels and the predicted pixels for each food category, divided by the union of these (1), (b) the accuracy of the segmentation, which is the intersection of GT_seg_ and the predicted pixels divided by the number of GT_seg_ pixels for each food category (2), (c) the $f_{min}^{t=>s}$ index (3) which represents the worst food category performance, and (d) $f_{sum}^{t=>s}$, which represents the average food category performance (4), from a segmentation *S* to a segmentation *T* (*S* and *T* are the GT_seg_ and the predicted segmentation masks). Each index is used in both directions (from *S* to *T* and from *T* to *S*) to estimate the total $F_{min}$ and $F_{sum}$ (5), which are the harmonic means of the minimum and the average indexes, respectively.

(1) $$IoU=\frac{gt\cap pred}{gt\cup pred},$$

(2) $$Accuracy:\;\frac{gt\cap pred}{gt},$$

(3) $$f_{min}^{t=>s}=Min_{i}\left(\frac{Max\left(\left|S_{i}\cap T_{j}\right|\right)}{\left|S_{i}\right|}\right),$$

(4) $$f_{sum}^{t=>s}=\frac{\sum_{i}Max_{j}\left(\left|S_{i}\cap T_{j}\right|\right)}{\sum_{i}\left|S_{i}\right|},$$

(5) $$F_{x}=\frac{2*f_{x}^{t=>s}*f_{x}^{s=>t}}{f_{x}^{t=>s}+f_{x}^{s=>t}},$$

### 2.4. Automatic Volume and Macronutrient Estimation

**Figure 6:**
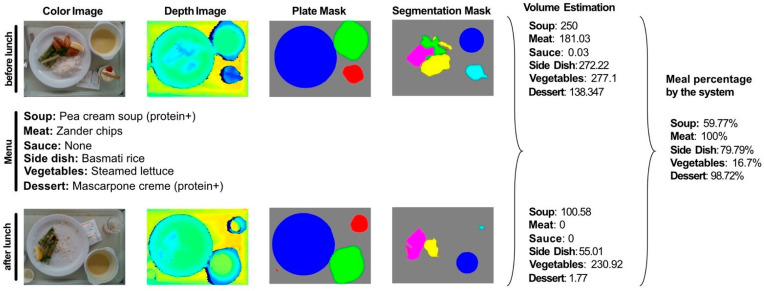
The system receives as input the daily menu, the RGB-D images, and the plate and meal segmentation masks and estimates the volume of each dish before and after consumption.

In order to estimate the volume that was consumed for each meal and, thus, estimate the macronutrient intake, we followed the algorithm that was used in our previous work [24,30]. For each meal, the menu of the day, the RGB image, the depth image, and the plate and food segmentation masks, which were provided by the segmentation network, were required for both the “before” and the “after” consumption images. The depth image was used to create the 3D point cloud, which was used to construct the food and the plate surface, based on the segmentation masks. The volume for each food category was then estimated by subtracting the food and the plate surface. The percentage consumed of each dish was calculated from the volume estimations of the “before” and the “after” meals, as shown in Figure 6. Finally, for each dish, the percentage of the meal that was consumed was multiplied by its nutritional value, retrieved from the kitchen database, in order to calculate the total energy and macronutrient intake. It is worth noting here that the calorie and macronutrient intake of the sauce was discarded, following the same estimation procedure as the medical student and the dietitians.

## 3. Results

### 3.1. Food Segmentation

Table 2 summarizes a comparison between the metrics for the different network architectures. When trained only on the new data, the PSPNet, with the simple encoder as its backbone gives the best results for all metrics except for the $F_{sum}$, which is slightly worse than with the ResNet + PSPNet architectures. It is also apparent that adding the plate module in parallel with the food segmentation module leads to better results for the food segmentation as well. Moreover, the PSPNet architecture seems to perform better than the DeepLabv3 network in almost all cases.

Finally, the same experiments were conducted while also using the meal images from our previous study [24] to evaluate the effect of using data from another hospital environment in the pretraining of the network. The 534 meal images that were gathered for training and testing the network of our previous study were used to pre-train the network for 20 epochs, before its fine-tuning on the data from the geriatric clinic of St. Gallen for 80 more epochs. Small adjustments were made to the data from the previous study in order to be consistent with the newly acquired data. The vegetables and salad, which were considered as different categories in our previous study [24], were merged into one, while the packaged containers were discarded. By doing that, although the Encoder + PSPNet achieved slightly worse results, the ResNet + PSPNet had a much better performance. Specifically, it achieved a mean Intersection over Union (*IoU*) of 73.7%, mean accuracy of 84.1%, and *F_min_* and *F_sum_* of 69.8% and 93.4%, respectively, outperforming all other network architectures. Even though the Encoder + PSPNet had better results originally when it was trained only on the newly acquired data, the network, due to its shallower architecture, could not generalise from one dataset to another. On the other hand, the deep network ResNet + PSPNet was able to find the connections between the two datasets and achieved better results.

**Figure 7:**
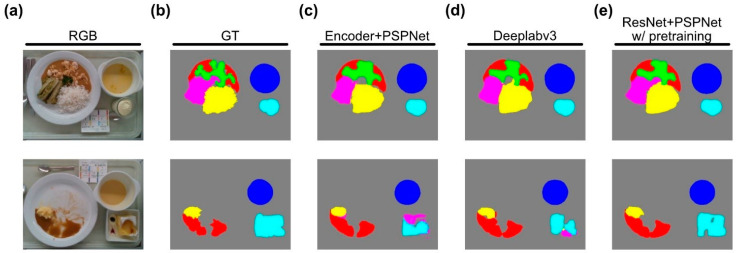
Segmentation results using different architectures: (**a**) the original RGB image; (**b**) the GT segmentation mask (GT_seg_); (**c**) the segmentation mask from the Encoder + PSPNet; (**d**) the segmentation mask from DeepLabv3; (**e**) the segmentation mask from ResNet + PSPNet w/pretraining.

Figure 7 also shows the comparison between the segmentation results of the Deeplabv3, the Encoder + PSPNet, and the ResNet + PSPNet with (w/) pretraining network architectures (with the plate module) for two sample images. The background is shown in grey, the soup in blue, the meat/fish dish in green, the side dish in yellow, the sauce in red, the salad in pink, and the dessert in light blue. Even though the output segmentation masks for the first exemplary image are quite similar for the different architectures, the masks for the second exemplary image prove the point that the ResNet + PSPNet w/pretraining outperforms the other networks. It is the only network that accurately predicts the dessert, without confusing it with the other categories; therefore, we use the ResNet + PSPNet w/pretraining as the segmentation network of our system.

### 3.2. Macronutrient Estimation Assessment

**Figure 8:**
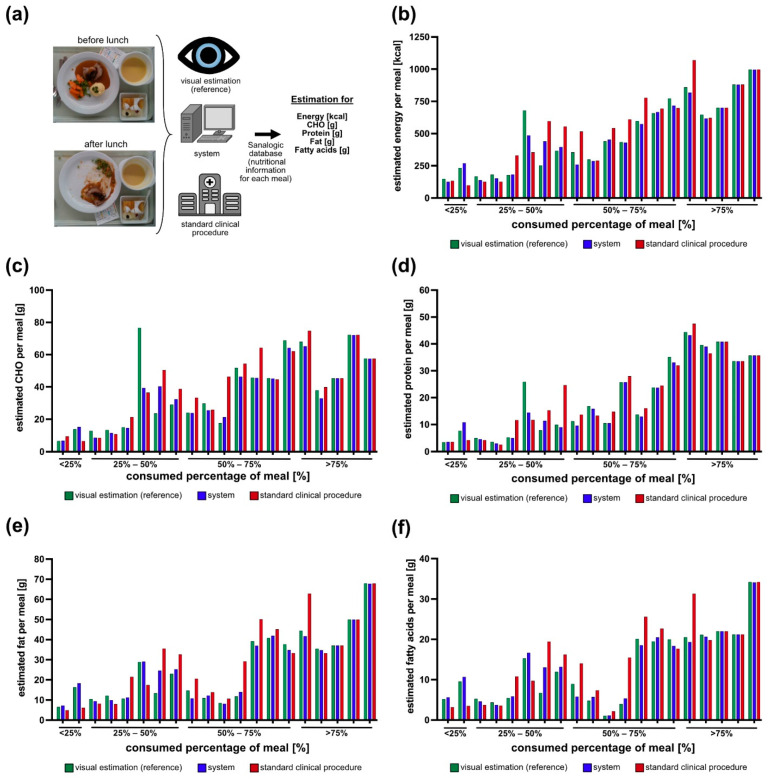
Bar plots for the 20 testing meals ordered by consumed percentage of each meal. (**a**) Schematic depiction of the workflow. Estimations for the (**b**) energy (kcal); (**c**) CHO (g); (**d**) protein (g); (**e**) fat (g); (**f**) fatty acids (g) intake. The green bars indicate the visual estimations of the dietitians and the student (reference), the blue bars indicate the system’s estimations, and the red bars the nursing staff following the standard clinical procedure.

**Figure 9:**
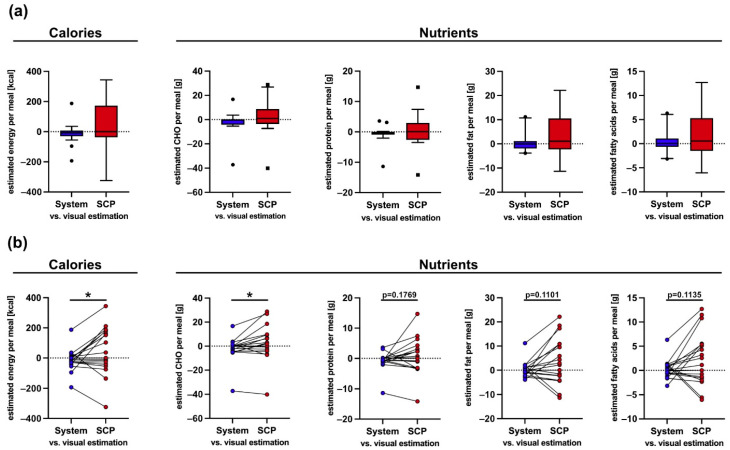
Differences in calories and macronutrient estimations per meal for the estimation methods. Difference was calculated by subtraction of the estimated calories or macronutrients of the system or the standard clinical procedure (SCP) from the visual estimation (reference) for each meal. Variation between the different estimations depicted as a box-plot in (**a**), where the circle and the square indicate the outliers, or as paired individual values in (**b**). The visual estimation is shown as a dotted line at a value of 0. Statistical analysis was performed using a paired-*t*-test. * = *p* ≤ 0.05.

In order to assess the energy and macronutrient intake as estimated by the system, we compared its results with those of the nursing staff following the standard clinical procedure (SCP) against the visual estimations of the two dietitians and the trained medical student for the 10% scale, which is considered as the reference (Figure 8a). For the segmentation network, we chose the ResNet + PSPNet w/pretraining since it gave the best segmentation results (Table 2 and Figure 7). The testing set consisted of the 20 meals that were also used for the evaluation of the segmentation network. For the nurses’ and the visual estimations, the energy and macronutrient intake of the patients were calculated on the basis of the daily menu for each of the 20 testing meals (Figure 8b–f). The SCP (red bars) were, in most cases, further apart from the visual estimations (green) and the system (blue). For the meals fully consumed, the nurses, the system, and the two dietitians and medical students all predicted that nearly 100% of the meal was consumed, and therefore, their estimation is the same. In comparison to the visual estimations and the system, the estimations from the SCP showed a wider distribution. Most often, the SCP overestimated the energy and macronutrient intake of the respective meals (Figure 9a). In a direct comparison of the system and SCP estimations for each meal, the system showed a significantly better performance for energy and CHO estimation, while it showed a trend towards significance for protein, fat, and fatty acid estimation (Figure 9b).

To quantitively analyse the results, for each meal, the mean of the two dietitians’ and the trained medical student’s estimations were considered as the reference, and we compared these results with those of the nurse and the AI system in terms of Mean Absolute Error (MAE) and Mean Relative Error (MRE) (Table 3). The results show that the system estimated the macronutrient and energy intake better than the nursing staff (error for the system: <15% for macronutrients and 11.64% for energy, error for nursing staff: >30% for macronutrients, 31.45% for energy)—who followed the clinic’s typical procedure. The correlation between the system’s estimations and the visual estimations (reference) is also very strong (r > 0.9, *p* < 0.001) and higher than that of the nursing staff. This is also underpinned by the percentage error of each individual component of each dish for estimations of the system and the nurse (Table 4). Moreover, the system can output the results almost at once, while the nursing personnel needed to first perform the estimation of the consumed food, find the respective menu in the database, and calculate the total energy and macronutrient intake for each patient; therefore, the usage of the system can provide accurate results, with high comfort.

## 4. Discussion

In this study, we assessed the performance of an end-to-end system to evaluate the macronutrient intake of patients in an acute geriatric hospital. Since the GT was not obtained by weighing the dishes and the meal components, two dietitians and one trained medical student estimated the percentage that was consumed from each food component in every meal, and these estimations were considered as the reference, as this method gives estimations as close to the GT as possible. On the other hand, the nurses followed the standard procedure of estimating the percentage that was eaten from the whole meal. On the basis of these estimations and the respective macronutrient information retrieved from the kitchen database, the energy and macronutrient intake of the patients for the meal was calculated. For the energy calculation, the system’s estimations had a mean error of only 41 kcal (11.64%) per meal, while the nursing staff’s estimation had an error of 112 kcal (31.45%). For the carbohydrate and the protein intake, the errors were, respectively, 4.6 g (13.23%) and 1.4 g (10.47%) for the system and 9 g (33.88%) and 3.7 g (34.34%) for the SCP. For the fat and fatty acids intake, the mean errors were 1.9 g (11.70%) and 1.2 g (14.84%), respectively, for the system, while for the SCP the errors were 7 g (41.29%) and 4.1 g (56.42%), which means more than three times the system’s errors.

In older hospitalised patients, the overall energy and protein intake are critical factors to prevent malnutrition [3,8]; therefore, the large mean SCP error for the energy and the protein intake combined with the fact that the nursing staff more often overestimated the energy and protein intake of the respective patient (Figure 9a), could lead to the wrong impression that the patient’s energy and protein intake was sufficient. In contrast, the system’s estimations differed only minimally from the visual estimations (reference) (Figure 8b–e and Figure 9a,b), which is reasonable since the dietitians and the medical student provided the results for every component of the meal separately, while the SCP estimated the total consumed food; therefore, the usage of the system in the hospital could lead to better identification of malnutrition and monitoring of the energy and macronutrient intake of patients, especially for those that do not consume their entire meal.

In addition to the macronutrient intake, we also estimated the accuracy of our results for every meal component in terms of the consumed percentage. For the estimations of the system, the lowest error appeared for the meat/fish component (6.56%), which could be more easily distinguished by the algorithm than the other food components on the plates, and for the salad/vegetables (7.46%). Although soup was always served in a separate soup bowl, which was easy to be segmented, the mean error was slightly higher (8.08%) because the depth estimation was not always accurate due to its reflective surface. The estimations of the side dishes and the dessert ranked next, with mean errors of 9.50% and 10.74%, respectively. All the SCP estimations had a mean error of at least 10% higher than the mean error of the system, apart from the side dish, for which the results were quite similar (9.50% for the system vs. 12.67% for the nurse). Interestingly, the greatest SCP estimation errors occurred for the soup and the dessert, which were the components with the highest energy and protein content, as they were often enriched with protein powder. The size error can partially be explained by the fact that during SCP several different people were involved, while the algorithm remains identical for each patient. Since energy and protein intake in older patients is prescribed to prevent and address under- or malnourishment [7,8], their inaccurate estimations could make difficult both the detection of malnutrition and also its treatment in the clinics; therefore, the integration of our system, which better estimated the total energy and macronutrient intake, could provide a solution to the aforementioned problem.

In comparison to other studies that make use of AI-based methods to assess calorie and macronutrient intake in a specific environment, our study provides better results. More specifically, in the current work, the system achieved slightly higher performance with respect to the MRE for both energy and macronutrients estimations compared to [24]. The improvement can be explained because of the less complex setup. In the current study, we had six food components and four plate types and we are using data to pretrain the segmentation network and gain information from the kitchen’s menus. In our previous study, we had seven food components and five plate types, there was no pretraining, and the meals had to be weighed. Furthermore, in [31], the authors use a system with a 3D scanning sensor on top of a smartphone to segment and estimate the volume of the food components and then calculate the nutritional intake based on the database of a hospital’s kitchen. In their study, twelve participants split into groups with different levels of knowledge regarding the meal (given the exact recipe, given a list of recipes, no information on the recipe) were recruited to evaluate the system’s performance. Even though the system’s estimations were better than those of the 24-h recall method and a web application, the mean error for the total volume of the meals was 33%. However, it must be noted that a head-to-head comparison of the quantitative results of this study with results from other studies is difficult since there is a lack of publicly available food tray datasets that also include depth images, nutritional information for each meal as well as their corresponding recipes. Finally, the menus that were used were unique for the specific hospitals and were known a priori, and thus, gathering more data, which could have a positive effect on the results, was not feasible.

One limitation of our study is that we did not use the real GT, by measuring the weights of the meals, as the process can be extremely time-consuming and effort demanding. All the different meals/dishes would need to be served on different plates, and the weight of each plate should be measured before and after consumption. This was part of our previous investigation [24], where the results of the system were compared to the real GT. In addition, we did not collect clinical data of the studied patients; therefore, we were not able to correlate the calculation of the system with any disease.

Our newly developed AI-based system for the estimation of the consumed food of patients offers better accuracy than the standard clinical procedure used in the respective clinic; therefore, the regular usage of our system in the hospital could lead to a better diagnosis of under- and malnourished patients and could also help with the tracking of the calorie and macronutrient intake of the patients. The use of our system could contribute to addressing the open issue of malnutrition in an acute hospital by providing an automatic, real-time, and more accurate than the standard clinical procedure dietary assessment method. Furthermore, the consequent use of such a system might allow a much more individualised adaptation of nutritional supplements based on the results of the analysis. Additionally, our system offers an effort- and time-saving method for the tracking of the consumed calories and macronutrients of patients. The AI-based system can be used on a computer or tablet, with very low computational needs. The whole process of segmenting the food image, linking with the kitchen database, and performing energy and macronutrient estimation is fully automated, and takes approximately 1 s to be completed. Further, no staff is needed to undertake the arduous process of portion size estimation, identifying food items, and entering the results. The only time required is a total of ~15 s to capture the images of the meal before and after it was consumed. By accurately estimating the caloric and macronutrient content of the provided and consumed food, maintenance of good nutritional status during hospitalisation may be optimised. Thus, additional hospitalisation time will be avoided, which is usually associated with high costs and the draining of rare hospital resources in times of hospital bed shortages. Since one of the main reasons that food protocols are hardly integrated into the daily clinical practice is the high time effort of the process of the estimation [23], the implementation of our system could increase the usage of food protocols in the daily clinical practice leading to the increased recognition of malnutrition in patients. Lastly, by providing accurate and reliable data, an archive of consistent and complete nutrient information for each patient will be created, and the collected data will be used for the assessment of long-term nutritional status, including information on how to handle malnutrition after discharge.

The system can be easily adapted to any new hospital or long-term care environment, as long as a hospital kitchen management system is used. Nevertheless, we recommend a small amount of training data (~300 images of meals) so that the segmentation-recognition network provides sufficient results. In order to collect the data and do the visual estimation, the medical student had to work approximately 1.5 h per day for 32 days. Adding more meal images, even from a different environment, can further improve the accuracy of the results. Additional costs required for such a system include the semi-automatic annotation of the training images (~2.5 min per image) and the training of healthcare providers to efficiently use the RGB-D camera for the image acquisition. However, compared to the SCP our system can provide results in real-time without the need for visual estimations.

For future work, we plan to integrate a barcode reader into the system to include information for the intake of packaged products, as well as a patient’s tray QR code reader for explicit patient identification. The latter allows the creation of a personal profile containing information about the daily and weekly nutritional intake along with suggestions for a more balanced diet. Emphasis will also be given to the ease-of-use and user-friendliness of the system, and a cost–benefit analysis will be conducted. The development of the communication interfaces with the kitchen management system for the automatic data exchange and the clinical validation of the integrated system are in the pipeline as part of our research and development work in the field.

## 5. Conclusions

In this paper, we presented an automatic, end-to-end, AI-based system that receives as input RGB-D food images captured on a standardised mount before and after consumption, along with the daily menu of the clinic’s kitchen, and is able to estimate the patients’ energy and macronutrient intake. The system consists of a segmentation network, which is trained to segment the different food types and plates, and a volume/macronutrient estimation module that evaluates the volume of each food type before and after consumption. The percentage consumed from each meal component was then linked with the kitchen’s database in order to calculate the total energy and macronutrient intake for each patient. The system offers high accuracy, provides the results in an automatic manner, and holds the potential to lower the cost of dietary assessment in a hospitalised environment; therefore, the system could contribute towards enhanced dietary monitoring and assessment of hospitalised patients at risk of malnutrition. Further clinical trials using the system to evaluate its potential in terms of a more individualised malnutrition management in geriatric institutions are necessary.

## Figures and Tables

**Table 1:** The lunch menu of the kitchen for one day, which contains three slightly different meals.

Meal	Soup	Meat/Fish	Side Dish	Sauce	Salad	Dessert
A	Potato and carrot cream soup (protein+)	Trout fillet	Potato wedges	/	Creamed spinach	Pineapple mousse (protein+)
B	Potato and carrot cream soup (protein+)	/	Pappardelle noodles (soft homogenous)	Bolognaise Sauce	/	Pineapple mousse (protein+)
C	Potato and carrot cream soup (protein+)	Trout fillet	Potato wedges	/	Creamed spinach	/

**Table 2:** The segmentation results for the different network architectures.

Segmentation Network	Mean Intersection over Union (%)	Accuracy (%)	*F_min_* (%)	*F_sum_* (%)
DeepLabv3	70.2	80.3	65.1	92.4
DeepLabv3 w/plates	70.8	80.8	65.2	92.5
ResNet + PSPNet	70.4	80.2	65.2	92.7
ResNet + PSPNet w/plates	70.7	81.1	65.5	92.9
Encoder + PSPNet w/plates	71.9	81.6	69.3	92.7
Encoder + PSPNet w/plates w/pretraining	69.7	78.6	68.2	92.1
ResNet + PSPNet w/plates w/pretraining	73.7	84.1	69.8	93.4

**Table 3:** Comparative results of the system and the nurse for the energy and the macronutrient intake for the testing set (*p* < 0.001).

	System			Standard Clinical Procedure (SCP)		
	Mean Absolute Error (Standard Deviation)	Mean Relative Error %	Correlation Coefficient	Mean Absolute Error (Standard Deviation)	Mean Relative Error %	Correlation Coefficient
Energy (kcal)	41 (54)	11.64	0.967	112 (102)	31.45	0.861
CHO (g)	4.6 (8.3)	13.23	0.905	9.0 (10.8)	33.88	0.790
Protein (g)	1.4 (2.5)	10.47	0.979	3.7 (4.1)	32.34	0.919
Fat (g)	1.9 (2.4)	11.70	0.984	7.0 (6.4)	41.29	0.877
Fatty acids (g)	1.2 (1.4)	14.84	0.978	4.1 (3.7)	56.42	0.841

**Table 4:** Comparative results between the system and the nurse for consumed percentage for each food category.

	System Error (%)	Standard Clinical Procedure Error (%)
Soup	8.08	24.04
Side dish	9.50	12.67
Meat/fish	6.56	19.61
Salad/vegetables	7.46	21.50
Dessert	10.74	34.67

## Data Availability

The datasets generated during and/or analyzed during the current study are not publicly available. The data may be however available upon reasonable request from the corresponding author.
